# C-reactive protein is associated with pain-type somatic symptoms independent of mental health symptoms in adolescents: Evidence from the ALSPAC study

**DOI:** 10.1016/j.bbih.2025.101082

**Published:** 2025-08-05

**Authors:** J. Cooney-Quane, D.S. Thomas, Y.M. Nolan, S. Dockray

**Affiliations:** a School of Applied Psychology, Cork Enterprise Centre, University College Cork, Cork, Ireland; b Department of Anatomy and Neuroscience, Western Gateway Building, University College Cork, Cork, Ireland

**Keywords:** Systemic inflammation, Mental health, Anxiety, Depression, Somatic pain symptoms, Adolescence, Psychoneuroimmunology

## Abstract

Depression and anxiety disorders frequently first present during adolescence, and both conditions are often comorbid with the experience of pain-type somatic symptoms. Moreover, increased concentrations of blood-derived inflammatory markers, such as C-reactive protein (CRP), have been observed in both depression and anxiety. Altered neuroimmune activation may impact on pain signalling pathways in the nervous system, potentially playing a role in the relationship between mental health and pain-type somatic symptoms.

This study conducted cross-sectional secondary data analyses of the Avon Longitudinal Study of Parents and Children (ALSPAC) dataset, using a sample of 2877 participants at age 18. Baron and Kenny's (1986) mediation framework was used to explore whether CRP acts as a mediator between depression and anxiety scores, and pain-type somatic symptoms. While CRP cannot be said to directly mediate the relationship in this sample, adjusted regression analysis found that CRP was a significant, independent predictor of pain-type somatic symptoms (*β* = .12, p < .001), independent of anxiety score (*β* = .20, p < .001), depression score (*β* = .38, p < .001), and the interaction term anxiety∗depression (*β* = −.15, p < .001), indicating that CRP may underly pain-type somatic symptoms, independent of mental health symptoms in adolescence.

These findings highlight the potential role of inflammatory processes in adolescent pain, and suggest that future research should examine biological factors, including inflammatory markers not typically assessed in clinical settings, that could underly pain symptoms not fully explained by mental health.

## Highlights

• Cross-sectional analysis at age 18 of CRP as a mediator between anxiety and/ore depression and pain-type somatic symptoms.
• Pain-type somatic symptoms of anxiety and depression: stomach, joint, chest, throat, neck/armpit gland pain, and headaches.
• While CRP did not mediate the relationship, it was a significant independent predictor of pain-type somatic symptoms.
• Further study of biological pain causes (e.g. inflammation) is needed when symptoms may be viewed as mental health related.

## Teaser

PerspectiveThis study provides insight into potential underlying pathophysiological process of pain-type somatic symptoms, by showing that C-reactive protein may be associated with pain-type somatic symptoms, independent of anxiety or depression score. These findings highlight the importance of further research into the immunological contributors to pain symptoms, especially when these pain symptoms are thought to be “medically unexplained”, to reduce the risk of diagnostic overshadowing and misdiagnosis.

## 1 Introduction

Depression and anxiety disorders often first present during adolescence and early adulthood, (Beesdo et al., 2009; Kessler et al., 2005b, Kessler et al., 2005a; McGrath et al., 2023; Solmi et al., 2021), early onset is associated with higher levels of disorder severity and symptom recurrence (Campbell et al., 2003; Herzog et al., 2021). In addition, depression and anxiety disorders are frequently comorbid (Kessler et al., 2015). Anxiety and depression have heterogenous presentations (Buch and Liston, 2021; Drzewiecki and Fox, 2024), yet vegetative (e.g. fatigue, changes in appetite) and somatic symptoms (e.g. pain, such as headaches, stomachache) may be more common in children and adolescents with depression, compared to adults (Rice et al., 2019).

In psychiatry and cognate disciplines, the characterization of somatic symptoms is often ambiguous and inconsistent, and may include pain, fatigue (although fatigue is sometimes categorized as a vegetative symptom; Rice et al., 2019), dizziness, and digestive disturbances. While somatic symptoms are commonly experienced by individuals with anxiety and depression, they are often medically unexplained or only partially attributable to identifiable physiological causes (Henningsen et al., 2003; Kroenke, 2003). Furthermore, many widely-used psychometric measures of anxiety and depression, such as the Beck Depression Inventory (Beck et al., 1996), Hamilton Depression Rating Scale (Hamilton, 1960) and Beck Anxiety Inventory (Beck et al., 1988), include multiple somatic symptom items (Wagner et al., 2012), underscoring how somatic symptoms are common in these conditions, but are also central to how these conditions are conceptualized and assessed (Kapfhammer, 2006; Maj et al., 2020). These somatic symptoms are increasingly understood to likely be driven by underlying physical health conditions, or by changes in the hypothalamic-pituitary-adrenal axis, changes in neuronal signaling in the vagus nerve and neural pain pathways, or low-grade systemic inflammation, for example elevated c-reactive protein (CRP) and pro-inflammatory cytokine levels (Iob et al., 2020; Liu et al., 2019; Frank et al., 2021).

Previous research has supported a link between peripheral inflammation and mental health conditions such as depression, anxiety and bipolar disorder (e.g. Dowlati et al., 2010; Howren et al., 2009; Costello et al., 2019; Solmi et al., 2021, Sayana et al., 2017; Upthegrove and Khandaker, 2020) however, findings are mixed, possibly due to the heterogeneous nature of these conditons and their symptoms. While less research has explored the relationship between comorbid anxiety and depression, there appear to be increases in some inflammatory markers for those with comorbidity (Shim et al., 2016; Wang et al., 2022). While much of the research has observed that inflammation precedes depression (Osimo et al., 2021; Valkanova et al., 2013), there is some evidence to suggest that the relationship may be bidirectional (Huang et al., 2019; Mac Giollabhui et al., 2021), and that depression (Lamers et al., 2019), anxiety, and stress (Muscatell et al., 2015; Steptoe et al., 2007) may precede inflammation. C-reactive protein (CRP) is an inflammatory protein which, along with pro-inflammatory cytokines, has been associated with immune-mediated depression and anxiety (Costello et al., 2019; Colasanto et al., 2020).

Positive associations between CRP and interleukin-6 (IL-6; a proinflammatory cytokine), and somatic depression symptoms have been observed in adults and children (Chu et al., 2023; Frank et al., 2021). In addition, CRP and the proinflammatory cytokines interleukin-1 (IL-1), IL-6, tumor necrosis factor-alpha (TNF-α), and interleukin-1 beta (IL-1β) have been associated with pain (Farrell et al., 2024; Ronchetti et al., 2017; Sommer and Kress, 2004; Strouse, 2007). Cytokines may directly influence pain through direct effects on neuronal signaling in the peripheral and central nervous system (Shubayev et al., 2010; Sommer and Kress, 2004; Vergne-Salle and Bertin, 2021), and indirectly by influencing other chemicals which play a role in pain signaling (e.g. nitrous oxide; Zhang and An, 2007). CRP synthesis is triggered by increased concentrations of systemic pro-inflammatory cytokines, and thus, as a downstream marker of pro-inflammatory cytokine activation, is a standard measure of peripheral inflammation (Ali et al., 2023; Mantovani and Garlanda, 2023).

The current study focuses on a specific subclass of somatic symptoms, referred to here as pain-type somatic symptoms. These are physical symptoms characterized primarily by experiences of bodily pain, and thought to be associated with, or commonly co-occur alongside anxiety and depression. The current study hypothesizes that biological changes underpin these pain-type somatic symptoms, and that these changes may not be fully recognized or accounted for in psychiatry and cognate fields of clinical practice. To this end, there is a risk that some such symptoms may be misattributed solely to psychological distress, a bias known as diagnostic overshadowing (Jones et al., 2008; Molloy et al., 2023), leading to the under-recognition of potential underlying biological contributors or health conditions. By conceptualizing CRP as a mediator, we move beyond describing the association between mental health and pain-type somatic symptoms, offering a biologically plausible mechanism which may explain the co-occurrence of psychological distress and pain-type somatic symptoms, or potentially identify CRP as an independent contributor to pain symptoms. By analysing the relationship cross-sectionally, this study seeks to extend the literature which suggests that psychological distress is associated with inflammatory responses, and which may also be associated with pain experience. Using cross-sectional data collected when participants were aged 18 years, the aim of this study is to apply a mediation model to assess if CRP directly mediates any relationship between pain-type somatic symptoms and anxiety and/or depression during late adolescence.

## 2 Methods

### 2.1 Sample

The Avon Longitudinal Study of Parents and Children (ALSPAC) is an ongoing multi-generational cohort study that enrolled pregnant women with expected delivery dates between April 1, 1991 and December 31, 1992, and living in and around Bristol, UK. 14,541 pregnant women were recruited at this time. These women and their partners completed questionnaires at regular intervals during pregnancy, and post-partum regarding themselves and their offspring. When the offspring cohort were approximately 7 years old, additional families with eligible children born during the original recruitment period were enrolled in the study. From age 7 years onwards, the offspring cohort attended annual clinic assessments. Data were collected from parents and their children on a wide range of topics, including demographics, physical health, mental health, lifestyle factors, and life experiences. The current study uses data from the offspring sample. The ALSPAC sampling strategy and cohort profile is described at https://www.bristol.ac.uk/alspac/researchers/cohort-profile/

A total of 3287 participants provided blood samples and completed mental health and wellbeing assessments at age 17.5 years. 2339 participants completed all measures.

Ethical approval for the study was obtained from the ALSPAC Law and Ethics Committee and the Local Research Ethics Committees. Detailed information on the ALSPAC study is available on the study website (https://www.bristol.ac.uk/alspac/), which includes a fully searchable data dictionary (https://www.bristol.ac.uk/alspac/researchers/access/). Access to the data required for this study was approved by the ALSPAC Executive Committee, and secondary analysis of the data was approved by the Social Research Ethics Committee, University College Cork, Ireland.

### 2.2 Measures

#### 2.2.1 Mental health symptoms, age 17.5 years

Anxiety and Depression symptoms were assessed using the Computerised Interview Schedule- Revised (CIS-R) (Lewis, 1994). The CIS-R is a widely used, self-administered diagnostic instrument which can be used to assign International Classification of Diseases, 10th Revision (ICD-10) diagnoses of anxiety and depressive disorders, among others. It also provides anxiety and depression score sub-scales which are detailed below. In the current study, discrete continuous scores for depression and anxiety were used to capture variation in symptom severity, rather than for diagnostic purposes. This approach is consistent with previous research in population-based mental health, including studies with this participant cohort Chaplin et al. (2021); Khandaker et al. (2014); Lewis et al. (2020), and in adult cohorts (Bhui et al., 2011; Roberts et al., 2014). The CIS-R includes four items which are used to calculate Anxiety Score: “On how many of the past seven days have you felt generally anxious, nervous or tense?", "How unpleasant has your anxiety, nervousness or tension been in the past seven days?", "In the past seven days, when you've been anxious, nervous or tense, have you had any of the following symptoms?: (heart racing or pounding, hands sweating or shaking, feeling dizzy, difficulty getting breath, butterflies in your stomach, dry mouth)”, and "Have you felt anxious, nervous or tense for more than 3 h in total on any day in the past seven days?". For example, the item "On how many of the past seven days have you felt generally anxious, nervous or tense?", the responses provided were 1 = “None”, 2 = “Between one and three days”, and 3 = “Four or more days". If 3 (“Between one and three days”) was selected, this item was assigned a score of 1. The CIS-R sums the scores of the four anxiety items to provide an Anxiety Score, which ranges from 0 to 4.

The CIS-R includes questions on core symptoms of depression based on the ICD-10*:* depression, fatigue, concentration, sleep, and depressive thoughts. Each of these symptoms is scored from 0 to 4, except for depressive thoughts which is scored from 0 to 5. An example of one of the items in the depressive thoughts sub-scale is “In the past seven days have you on at least one occasion felt guilty or blamed yourself when things went wrong, even when it wasn't your fault?”. The responses provided are 1 = “Never”, 2 = "Only when it was my fault”, 3 = “Sometimes”, and 4 = “Often”. If 3 (“Sometimes”) or 4 (“Often”) were selected, this item was assigned a score of 1. Further details of the items of each symptoms is included in Appendix B in the supplementary material. The CIS-R sums these symptom scores to provide a total Depression Score, which ranges from 0 to 21. Further details regarding CIS-R items and scoring can be found in the CIS-R manual (Lewis and Pelosi, 1992).

An interaction term anxiety∗depression score was derived by standardizing the CIS-R anxiety and depression scores, and then multiplying the z-scores to explore their combined effect.

#### 2.2.2 Systemic CRP, age 17.5 years

Serum samples were obtained from 3287 participants and stored at −80 °C. High sensitivity CRP (hsCRP) was measured by automated particle-enhanced immunoturbidimetric assay (Roche UK, Welwyn Garden City, UK). hsCRP values ranged from .02 to 176.1 mg/l. As this study is exploring pain-type somatic symptoms, for which no clinical CRP cut-offs have been defined, we included participants with CRP levels >10 mg/L. Individuals above the 99th percentile for CRP (>18.61 mg/L) were omitted. While immunopsychiatry research frequently excludes participants above 10 mg/L (e.g. Chu et al., 2019; Khandaker et al., 2016), as values above this threshold may potentially denote acute infection, this study is exploring pain-type somatic symptoms for which no clinical CRP cut-offs have been defined. In addition, pain-type somatic symptoms may be linked not only to low-grade inflammation, but also more elevated levels of CRP. Excluding CRP values above the 99th percentile (18.61 mg/L) was chosen to strike a balance between removing likely outliers indicative of acute illness, while retaining individuals who may have slightly higher inflammatory profiles linked to pain symptoms. A total of 29 participants were excluded due to CRP values exceeding this cut-off. Further to this, a variable “infection present in the three weeks prior to the clinic visit” was included as a confounder.

#### 2.2.3 Pain-type somatic symptoms, age 17.5 years

Pain-type somatic symptoms were assessed using the CIS-R. The authors derived a “Pain-type Somatic Symptom Score” by summing the severity scores of 7 pain-type symptoms from the CIS-R Somatic Symptom scale (Lewis, 1994). These symptoms, assessed over the previous 7 days were: “Severity of Indigestion/Stomach Ache”, “Severity of Joint Pains”, “Severity of Muscle Pain”, “Severity of Headaches”, “Severity of Chest Pain”, “Severity of Throat Pain”, “Severity of Pain in Neck/Armpit Glands”. Pain-type Somatic Symptom Score ranged from 0 to 21.

#### 2.2.4 Confounding variables

Similar to previous literature, a range of potential confounding variables relevant to systemic inflammation and psychiatric conditions were included (e.g. Chu et al., 2019; Khandaker et al., 2016; Perry et al., 2019). Participant variables were collected at birth: sex, and ethnicity, and during the Teen Focus 4 clinic visit at age 17.5 years: body mass index (BMI), cigarette, alcohol and substance use, and infection during the 3 weeks preceding the clinic visit. The substance use variable was derived from binary yes/no variables on the use of substances during the three months preceding the clinic visit (including un-prescribed medications, cocaine, amphetamines, inhalants, sedatives or sleeping pills, hallucinogens, opioids, cannabis, and other substances), at age 17.5 years.

Parent variables included: parental socioeconomic status at 32 weeks gestation (father's social class, and mother's highest educational qualification), and mother's age at delivery. Father's social class was recorded according to the UK Office of National Statistics classification system, and divided into 7 categories (I, II, III non-manual, III manual, IV, V, and Armed Forces). Mother's highest education level was divided into 6 categories (none, CSE, Vocational, O Level, A Level, Degree). Mother's age at delivery was grouped into six categories (<20, 20–24, 25–29, 30–34, 35–49, 40+ years).

#### 2.2.5 Exclusion criteria

To minimise potential confounders, we removed all participants with bleeding/clotting disorders (n = 6), hypertension (n = 24), diabetes (n = 10), high cholesterol (n = 5) or vascular disease (n = 27) at age 17.5 years. We also excluded participants who met the criteria for psychotic disorder (n = 28), according to Psychosis Like Symptoms Questionnaire (PLIKS-Q; Horwood et al., 2008) at age 17.5 years. See Table C.1. in the Supplementary Material.

### 2.3 Statistical analysis

An a priori power analysis was conducted using G∗Power version 3.1.9.6 (Faul et al., 2007) for sample size estimation, based on data from a meta-analysis by Colasanto et al. (2020) examining the relationship between depression and CRP cross-sectionally among children and adolescents, which found an effect size of r = .12, considered to be small using Cohen's (1988) criteria. With a significance criterion of α = .05 and power = .80, the minimum sample size needed with this effect size is *N* = 1231 for linear multiple regression analysis. Thus, the sample size in the current study is adequate to test the study hypothesis.

Data analysis was carried out in SPSS, version 28 (IBM). Correlation analyses were carried out to examine the relationship between continuous variables. Logarithmic transformations of CRP and BMI were used due to their asymmetric distributions. However, as is frequently the case with psychometric measures in observational studies, some variables (e.g. Anxiety Score, Depression Score) were skewed and contained many zero observations, and as such transformations were not appropriate. To account for non-normal distributions in some of the variables Spearman's Correlations were carried out.

Discrete-continuous scores were used for anxiety and depression to explore the relationship between the severity of the anxiety and depressive symptoms and CRP, and pain-type somatic symptoms. Linear regression modelling was used to examine the relationships between anxiety score and depression score, CRP concentration, and pain-type somatic symptom score at age 17.5 years. The analyses used a mediation model (Baron and Kenny, 1986) to test whether CRP, as a measure of systemic inflammation, directly mediated any association between anxiety score and depression score and pain-type somatic symptoms at age 17.5 years. Baron and Kenny's (1986) mediation framework involves three sequential steps: first, examining if the independent variable (anxiety and/or depression score) significantly predict the dependent variable (pain-type somatic symptoms); second, examining if the independent variable predicts the proposed mediator (CRP); and third examining if the mediator predicts the outcome while controlling for the independent variable, with a corresponding reduction in the strength of the direct effect. Data was analysed cross-sectionally.

#### 2.3.1 Mediation model stage 1: association between somatic pain and mental health

Hierarchical linear regression was used to examine the relationship between pain-type somatic symptoms and anxiety score, depression score, and the interaction between anxiety and depression scores. Ethnicity, father's social class, mother's highest education level, and mother's age at delivery were entered into Step 1; BMI, alcohol consumption, daily smoking, and substance use, and infection during the 3 weeks preceding the clinic visit were entered into Step 2; and anxiety score, depression score, and anxiety∗depression were included in Step 3 of the regression model.

#### 2.3.2 Mediation model stage 2: association between systemic CRP and mental health

Hierarchical linear regression was used to examine the relationship between CRP and anxiety, depression, and the interaction between anxiety and depression. Ethnicity, father's social class, mother's highest education level, and mother's age at delivery were entered into Step 1; BMI, alcohol consumption, daily smoking, and substance use, and infection during the 3 weeks preceding the clinic visit were entered into Step 2; and anxiety score, depression score, and anxiety∗depression were included in Step 3 of the regression model.

#### 2.3.3 Mediation model stage 3: association between somatic pain, systemic CRP and mental health

Hierarchical linear regression was used to examine the relationship between pain-type somatic symptoms, CRP and anxiety, depression, and the interaction between anxiety and depression. Ethnicity, father's social class, mother's highest education level, and mother's age at delivery were entered into Step 1; BMI, alcohol consumption, daily smoking, and substance use, and infection during the 3 weeks preceding the clinic visit were entered into Step 2; anxiety score, depression score, and anxiety∗depression were included in Step 3; and CRP was included in Step 4 of the regression model.

#### 2.3.4 Assumption checks

Collinearity Diagnostics for all 3 regression models were assessed, Tolerance and VIF scores indicated that there was no multicollinearity between the variables. As some of the variables were not normally distributed, standardised residuals were visually examined via Normal P-P Plots and Scatterplots. Residuals appeared approximately normal, although several cases had standardised residuals of greater than 3.3 and less than −3.3, however, this is not uncommon in large samples. Leverage values were examined, and several cases exceeded the group-specific thresholds, suggesting potential influence based on predictor values, however, no cases exceeded a Cook's Distance of 1.0, indicating that no single case had undue influence on the overall model. Mahalanobis Distance was used to assess multivariate outliers based on the number of independent variables in each regression model. Selecting an alpha level of .001 according to Tabachnick and Fidell (2013), several cases exceeded the Critical Chi-Square values for the number of independent variables included in the regressions (regression 1 and 2 Χ^2^c(13) = 31.528; regression 3 Χ^2^c(14) = 36.123). To evaluate the potential influence of these multivariate outliers, the regression analyses were re-run with these cases excluded. The pattern of significant predictors remained consistent in Regressions 1 and 3, though some changes in beta coefficients and R values were observed. Notably, Regression 2, which was not significant in the original analysis, became statistically significant after removing multivariate outliers, suggesting that these outliers may have masked meaningful associations in the third block of variables. The results of the adjusted regressions are presented in Table 3, Table 4, Table 5, and results of the unadjusted regressions are included in Table D.1., D.2., and D.3. in the supplementary material for comparison.

### 2.4 Preregistration

This research was preregistered on OSF, and this can be found at https://doi.org/10.17605/OSF.IO/XVQ42. The original preregistration outlined plans to conduct regression analyses stratified by sex, however the number of complete cases available for the adjusted regression model would have substantially reduced the number of cases per model, which in turn would have limited the robustness and reliability of the estimates. To preserve statistical power and ensure stability of the adjusted analyses, it was decided to analyse the full sample as a whole. Sex was then included as a predictor variable in the regression analyses.

## 3 Results

### 3.1 Study participants

Of the 2877 participants eligible for inclusion in the study at age 17.5 years, 2339 participants completed all measures. The mean age of the study participants was 17.7 years. 48.2% were male, and 51.8% were female. 95.7% were white, 4.3% were non-white. Descriptive statistics for the overall sample, and stratified by sex are presented in Table 1.

**Table 1:** Descriptive statistics for males and females.

Characteristic	Total Sample	Males	Females
Participants*, n*	2877	1387	1490
Age, mean (SD), years	17.77 (.38)	17.76 (.37)	17.77 (.40)
Ethnicity *n* (%)			
White British,	2495 (95.7)	1213 (95.8)	1282 (95.6)
Other	112 (4.3)	53 (4.2)	59 (4.4)
Mother's Education,*n*(%)			
CSE	274 (10.4)	126 (9.9)	148 (10.9)
Vocational	182 (6.9)	97 (7.6)	85 (5.7)
O Level	860 (32.6)	390(30.6)	470 (34.5)
A Level	771 (29.2)	388 (30.4)	383(28.1)
Degree	551 (20.9)	275 (21.6)	276 (20.3)
Paternal Social Class,*n*(%)			
I	372 (15.3)	180 (15.2)	192 (15.3)
II	948 (38.9)	471 (39.9)	477 (38.0)
III (non-manual)	286 (11.7)	139 (11.8)	147 (11.7)
III (manual)	603 (24.8)	280 (23.7)	323 (25.7)
IV	178 (7.3)	90 (7.6)	88 (7.0)
V	48 (2.0)	20 (1.4)	28 (2.2)
Armed Forces	1 (.0)	1 (.1)	0 (.0)
Mother's Age at Delivery,*n*(*%*)			
Under 20 years	194 (6.7)	90 (6.5)	104 (7.0)
20–24 years	355 (12.3)	159 (11.5)	196 (13.2)
25–29 years	1014 (35.3)	489 (35.3)	525 (35.3)
30–34 years	928 (32.3)	465 (33.5)	463 (31.1)
35–39 years	343 (11.9)	164 (11.8)	179 (12.0)
40+ years	42 (1.5)	20 (1.4)	22 (1.5)
BMI, mean (*SD*) kg/m2	22.62 (3.78)^a^	22.47 (3.57)	22.76 (3.96)
CRP, mean (*SD*), mg/l	1.26 (2.04)	.98 (1.61)	1.52 (2.35)
Anxiety Score, mean (*SD*)	.26 (.72)^a^	.19 (.64)	.33 (.78)
Depression Score, mean (*SD*)	2.99 (3.78)^a^	2.28 (3.22)	3.66 (4.13)
Pain-type Somatic Symptom Score, mean (*SD*)	2.90 (2.56)^a^	2.46 (2.33)	3.33 (2.70)
Infection Present in Last 3 weeks, *n* (%)	397 (15.2)	149 (11.9)	234 (17.5)
Daily Smoking, mean (*SD*)	1.13 (3.82)	1.15 (4.00)	1.10 (3.64)
Alcohol Consumption, mean (*SD*)	5.00 (3.65)^a^	5.28 (3.84)	4.74 (3.44)
Substance Use, mean (*SD*)	.34 (.79)	.39 (.87)	.31 (.70)
Valid, n (listwise)	2339	1133	1206

Abbreviations: BMI, body mass index; CRP, C-reactive protein; SD, standard deviation.

a Mann-Whitney *U* test for sex is significant at the .05 level (2-tailed).

### 3.2 Correlation analysis

Both Pearson's and Spearman's correlation analyses were carried out to test for associations between variables before controlling for covariates. However, as several variables exhibited skewed distributions with a high proportion of zero values, Spearman's coefficients more accurately represent the relationships between variables, and as such Spearman's correlations are presented below in Table 2. Significant positive correlations of note include: anxiety score and pain-type somatic symptom score; depression score and pain-type somatic symptom score; CRP and depression score; CRP and pain-type somatic symptom score, and significant negative correlations of note include: CRP and anxiety∗depression.

**Table 2:** Spearman's correlations between variables.

	Sex	Anxiety Score	Depression Score	Mother's Highest Education Qualification	Mother's age at delivery	Pain-type Somatic Symptom Score	Substance Use (3months)	Daily Smoking	Alcohol Consumption	Paternal Social Class	CRP (log10)	BMI (log10)	Anxiety∗ Depression
Sex	–												
Anxiety Score	.12∗∗	–											
Depression Score	.19∗∗	.35∗∗	–										
Mother's Highest Education Qualification	−.03	−.04	<-.01	–									
Mother's age at delivery	−.03	.01	−.01	.32∗∗	–								
Pain-type Somatic Symptom Score	.17∗∗	.23∗∗	.41∗∗	−.02	<.01	–							
Substance Use (3months)	−.03	.05∗	.14∗∗	.08∗∗	.06∗∗	.10∗∗	–						
Daily Smoking	.01	.06∗∗	.10∗∗	−.12∗∗	−.09∗∗	.10∗∗	.34∗∗	–					
Alcohol Consumption	−.06∗∗	.01	.06∗∗	−.02	<.01	.11∗∗	.34∗∗	.22∗∗	–				
Paternal Social Class	.01	.01	.03	−.42∗∗	−.28∗∗	<.01	<-.01	.14∗∗	.05∗	–			
CRP (log10)	.17∗∗	−.02	.05∗∗	−.08∗∗	−.05∗	.17∗∗	<.01	.05∗	.07∗∗	.08∗∗	–		
BMI (log10)	.04	−.01	.02	−.07∗∗	−.10∗∗	.05∗∗	−.02	<-.01	.07∗∗	.07∗∗	.31∗∗	–	
Anxiety∗Depression	−.04∗	.25∗∗	−.44∗∗	−.02	.04	−.15∗∗	−.05∗	−.03	−.02	−.03	−.05∗	−.03	–

∗∗. Correlation is significant at the .01 level (2-tailed).

∗. Correlation is significant at the .05 level (2-tailed).

**Table 3:** Regressions of associations between mental health and pain-type somatic symptoms.

Variable	*B*	*95 % CI*	*SEB*	*β*	*df*	*F*	*R2*	*R2(adj)*	*R2 Δ*
Step 1									
(Constant)	2.27	[1.21, 3.32]	.54	<-.01∗∗∗					
Ethnicity	−.51	[-1.24, .21]	.37	−.03					
Sex	.71	[.49, .94]	.11	.15∗∗∗					
Paternal Social Class	<-.01	[-.10, .09]	.05	<-.01					
Mother's Highest Education	<-.01	[-.11, .10]	.06	<-.01					
Mother's Age at Delivery	−.01	[-.14, .12]	.07	<-.01					
					5, 1706	8.32	.02	.02	.02
Step 2									
(Constant)	1.82	[-.78, 4.42]	1.33	<-.01					
Ethnicity	−.40	[-1.11, .31]	.36	−.03					
Sex	.65	[.43, .87]	.11	.14∗∗∗					
Paternal Social Class	−.03	[-.13, .06]	.05	−.02					
Mother' Highest Education	−.04	[-.14, .07]	.05	−.02					
Mother's Age at Delivery	<-.01	[-.13, .12]	.06	<-.01					
BMI (log10)	2.00	[.29, 3.71]	.87	.05∗					
Alcohol Consumption	.06	[.03, .10]	.02	.09∗∗∗					
Substance Use (3months)	.14	[-.04, .33]	.09	.04					
Daily Smoking	.04	[-.01, .08]	.02	.04					
Infection (last 3 weeks)	−1.34	[-1.67, −1.02]	.17	−.19∗∗∗					
					5, 1701	15.25	.08	.08	.06
Step 3									
(Constant)	1.83	[-.58, 4.23]	1.23	<-.01					
Ethnicity	−.18	[-.83, .47]	.33	−.01					
Sex	.32	[.12, .53]	.10	.07∗∗					
Paternal Social Class	−.04	[-.13, .05]	.04	−.02					
Mother's Highest Education	−.04	[-.14, .06]	.05	−.02					
Mother's Age at Delivery	<-.01	[-.12, .11]	.06	<-.01					
BMI (log10)	1.48	[-.10, 3.06]	.81	.04					
Alcohol Consumption	.06	[.03, .09]	.02	.08∗∗∗					
Substance Use (3months)	.03	[-.14, .20]	.09	<.01					
Daily Smoking	.04	[-.01, .08]	.02	.04					
Infection (last 3 weeks)	−1.18	[-1.48, −.88]	.15	−.17∗∗∗					
Anxiety score	.87	[.50, 1.23]	.19	.19∗∗∗					
Depression Score	.28	[.24, .31]	.02	.38∗∗∗					
Anxiety∗Depression	−.09	[-.14, −.04]	.03	−.15∗∗∗					
					3, 1698	37.02	.22	.22	.14

∗p < .05.

∗∗p < .01.

∗∗∗p < .001∗^.^

CI = Confidence Interval for B.

**Table 4:** Regressions of associations between mental health and CRP.

Variable	*B*	*95 % CI*	*SE*	*β*	*df*	*F*	*R2*	*R2(adj)*	*R2 Δ*
Step 1									
(Constant)	−.45	[-.65, −.25]	.10	<-.01∗∗∗					
Ethnicity	−.04	[-.17, .10]	.07	−.01					
Sex	.18	[.13, .22]	.02	.19∗∗∗					
Paternal Social Class	.02	[.00, .04]	<.01	.05∗					
Mother's Highest Education	<.01	[-.01, .03]	.01	.01					
Mother's Age at Delivery	−.01	[-.04, .01]	.01	−.03					
					5, 1706	14.22	.04	.04	.04
Step 2									
(Constant)	−2.86	[-3.34, −2.38]	.24	<-.01∗∗∗					
Ethnicity	−.05	[-.18, .08]	.07	−.02					
Sex	.15	[.11, .20]	.02	.17∗∗∗					
Paternal Social Class	.01	[-.00, .03]	<.01	.04					
Mother's Highest Education	<.01	[-.01, .02]	.01	.01					
Mother's Age at Delivery	<-.01	[-.03, .02]	.01	<-.01					
BMI (log10)	2.10	[1.78, 2.42]	.16	.29∗∗∗					
Alcohol Consumption	<.01	[.00, .01]	<.01	.04					
Substance Use (3months)	<-.01	[-.04, .03]	.02	<-.01					
Daily Smoking	<.01	[-.01, .01]	<.01	.02					
Infection (last 3 weeks)	−.23	[-.29, −.17]	.03	−.17∗∗∗					
					5, 1701	32.66	.16	.16	.12
Step 3									
(Constant)	−2.83	[-3.31, −2.35]	.24	<-.01∗∗∗					
Ethnicity	−.05	[-.18, .08]	.07	−.02					
Sex	.16	[.12, .20]	.02	.17∗∗∗					
Paternal Social Class	.01	[-.00, .03]	<.01	.04					
Mother's Highest Education	<.01	[-.02, .02]	.01	<.01					
Mother's Age at Delivery	<-.01	[-.03, .02]	.01	<-.01					
BMI (log10)	2.09	[1.77, 2.40]	.16	.29∗∗∗					
Alcohol Consumption	<.01	[.00, .01]	<.01	.04					
Substance Use (3months)	<-.01	[-.04, .03]	.02	<-.01					
Daily Smoking	<.01	[-.01, .01]	<.01	.02					
Infection (last 3 weeks)	−.23	[-.29, −.17]	.03	−.17∗∗∗					
Anxiety score	−.05	[-.12, .03]	.04	−.05					
Depression Score	<.01	[-.01, .01]	<.01	<.01					
Anxiety∗Depression	<-.01	[-.01, .01]	<.01	−.02					
					3, 1698	25.85	.17	.16	<.01

∗p < .05.

∗∗p < .01.

∗∗∗p < .001, CI = Confidence Interval for *B*.

**Table 5:** *Regressions of Associations Between* pain-type somatic symptoms, CRP, and mental health.

Variable	*B*	*95 % CI*	*SE*	*β*	*df*	*F*	*R2*	*R2(adj)*	*R2 Δ*
Step 1									
(Constant)	2.27	[1.21, 3.32]	.54	<-.01∗∗∗					
Ethnicity	−.51	[-1.24, .21]	.37	−.03					
Sex	.71	[.49, .94]	.11	.15∗∗∗					
Paternal Social Class	<-.01	[-.10, .09]	.05	<-.01					
Mother's Highest Education	<-.01	[-.11, .10]	.06	<-.01					
Mother's Age at Delivery	−.01	[-.14, .12]	.07	<-.01					
					5, 1706	8.32	.02	.02	.02
Step 2									
(Constant)	1.82	[-.78, 4.42]	1.33	<-.01					
Ethnicity	−.40	[-1.11, .31]	.36	−.03					
Sex	.65	[.43, .87]	.11	.14∗∗∗					
Paternal Social Class	−.03	[-.13, .06]	.05	−.02					
Mother's Highest Education	−.04	[-.14, .07]	.05	−.02					
Mother's Age at Delivery	<-.01	[-.13, .12]	.06	<-.01					
BMI (log10)	2.00	[.29, 3.71]	.87	.05∗					
Alcohol Consumption	.06	[.03, .10]	.02	.09∗∗∗					
Substance Use (3months)	.14	[-.04, .33]	.09	.04					
Daily Smoking	.04	[-.01, .08]	.02	.04					
Infection (last 3 weeks)	−1.34	[-1.67, −1.02]	.17	−.19∗∗∗					
					5, 1701	15.25	.08	.08	.06
Step 3									
(Constant)	1.83	[-.58, 4.23]	1.23	<-.01					
Ethnicity	−.18	[-.83, .47]	.33	−.01					
Sex	.32	[.12, .53]	.10	.07∗∗					
Paternal Social Class	−.04	[-.13, .05]	.04	−.02					
Mother's Highest Education	−.04	[-.14, .06]	.05	−.02					
Mother's Age at Delivery	<-.01	[-.12, .11]	.06	<-.01					
BMI (log10)	1.48	[-.10, 3.06]	.81	.04					
Alcohol Consumption	.06	[.03, .09]	.02	.08∗∗∗					
Substance Use (3months)	.03	[-.14, .20]	.09	<.01					
Daily Smoking	.04	[-.01, .08]	.02	.04					
Infection (last 3 weeks)	−1.18	[-1.48, −.88]	.15	−.17∗∗∗					
Anxiety score	.87	[.50, 1.23]	.19	.19∗∗∗					
Depression Score	.28	[.24, .31]	.02	.38∗∗∗					
Anxiety∗Depression	−.09	[-.14, −.04]	.03	−.15∗∗∗					
					3, 1698	37.02	.22	.22	.14
Step 3									
(Constant)	3.65	[1.18, 6.13]	1.26	<-.01∗∗					
Ethnicity	−.15	[-.79, .50]	.33	<-.01					
Sex	.22	[.01, .43]	.11	.05∗					
Paternal Social Class	−.05	[-.14, .04]	.04	−.03					
Mother's Highest Education	−.04	[-.14, .06]	.05	−.02					
Mother's Age at Delivery	<-.01	[-.12, .11]	.06	<-.01					
BMI (log10)	.13	[-1.51, 1.78]	.84	<.01					
Alcohol Consumption	.05	[.02, .09]	.02	.08∗∗∗					
Substance Use (3months)	.03	[-.14, .20]	.09	<.01					
Daily Smoking	.03	[-.01, .07]	.02	.04					
Infection (last 3 weeks)	−1.03	[-1.34, −.73]	.15	−.15∗∗∗					
Anxiety score	.90	[.54, 1.26]	.18	.20∗∗∗					
Depression Score	.28	[.24, .31]	.02	.38∗∗∗					
Anxiety∗Depression	−.09	[-.14, −.04]	.03	−.15∗∗∗					
CRP (log10)	.64	[.41, .88]	.12	.12∗∗∗					
					1, 1697	36.97	.23	.23	.01

∗p < .05.

∗∗p < .01.

∗∗∗p < .001, CI = Confidence Interval for *B*.

### 3.3 Multiple regression analysis

#### 3.3.1 Mediation model stage 1: regressions of associations between mental health and pain-type somatic symptoms

For the first stage of the mediation model, the relationship between mental health and pain-type somatic symptoms was explored by conducting a hierarchical regression (Table 3). Demographic variables in Step 1 accounted for 2% of the variable in pain-type somatic symptoms (R^2^ = .2, *p* < .001), with sex the only significant predictor. Adding health and lifestyle factors in Step 2 increased the explained variance to 8% (R^2^Δ = .06, p < .001), with BMI, and alcohol consumption emerging as significant positive predictors, and recent infection emerging as a significant negative predictor. In Step 3 the inclusion of mental health variables resulted in a substantial increase in variance explained to 22% (R^2^Δ = .14, p < .001). All three mental health variables were significant, with depression score and anxiety score showing positive associations (*β* = .38, p < .001, *β* = .19, p < .001, respectively), and the interaction term anxiety∗depression showing a negative association (*β* = −.15, p < .001).

#### 3.3.2 Mediation model stage 2: regressions of associations between mental health and CRP

For stage 2 of the mediation model, a hierarchical regression was conducted to examine predictors of the proposed mediator variable, CRP (Table 4). In Step 1, demographic factors explained 4% of the variance in CRP (R^2^ = .4, *p* < .001), with sex and paternal social class the only significant predictors. Adding health and lifestyle factors in Step 2 increased the explained variance to 16% (R^2^Δ = .012, p < .001), with BMI emerging as a significant positive predictor, and recent infection emerging as a significant negative predictor of CRP. In step 3, the inclusion of mental health variables resulted in a very minimal increase in explained variance (R^2^Δ = <.01, p < .04). None of the mental health variables were significant predictors of CRP.

#### *3.3.3* *Mediation model stage 3: regressions of associations between* pain-type somatic symptoms, CRP, and mental health

**Fig. 1:**
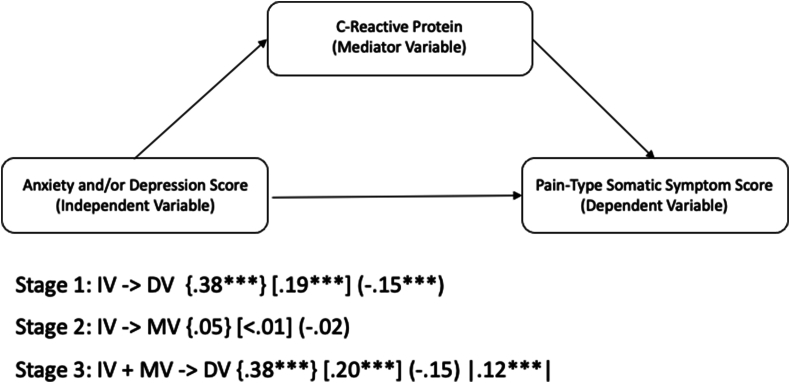
Mediation analysis results for mental health variables, CRP, and pain-type somatic symptoms. Note. Values represent standardized regression coefficients. Values for variables are presented as: {Depression Score}; [Anxiety Score]; (Anxiety∗Depression); |CRP| ∗∗∗p < .001.

The third and final stage of the mediation model was conducted to examine whether CRP accounted for any additional variance in pain-type somatic symptoms beyond the effects of demographic, health and lifestyle, and mental health variables (Table 5). CRP was entered in Step 4 of the hierarchical regression, and it accounted for a small, but statistically significant increase in explained variance (R^2^Δ = .01, p < .001), bringing the total R^2^ from 22% in Step 3, to 23% in Step 4. CRP was a significant independent positive predictor of pain-type somatic symptoms (*β* = .12, p < .001). The strength and significance of the mental health predictors remained largely unchanged following the inclusion of CRP in Step 4. Fig. 1 illustrates the results of the mediation analysis for mental health variables, CRP, and pain-type somatic symptoms.

## 4 Discussion

To our knowledge, this is the first study to examine whether CRP statistically mediates the relationship between anxiety and/or depression and pain-type somatic symptoms in adolescence, and finds that anxiety and depression scores, and their interaction, significantly predicted pain-type somatic symptom score and that CRP was a significant independent predictor of pain-type somatic symptom score, in addition to the mental health variables. However, neither anxiety score, depression score, nor the interaction term anxiety∗depression contributed to variance in CRP level. This pattern of associations does not meet the assumptions required for mediation (Baron and Kenny's 1986) and so CRP is not evidenced to mediate the relationship between mental health and pain-type somatic symptoms in this sample. Rather, CRP appears to act as an independent predictor of pain-type somatic symptoms, contributing additional explanatory power to the model, without altering the strength nor significance of the mental health predictors.

### 4.1 Mental health symptoms and CRP

The current study observed no association between mental health symptoms and CRP in regression analyses. Low-grade systemic inflammation has been observed to be associated with depression (Dowlati et al., 2010; Howren et al., 2009; Mac Giollabhui et al., 2021; Osimo et al., 2019), and with anxiety (Costello et al., 2019), including among adolescent populations (Khandaker et al., 2016; Toenders et al., 2022), albeit with small effect sizes. There are inconsistent findings however, and a meta-analysis by Parsons et al. (2021) found no associations between anxiety and inflammatory markers in children and adolescents, although there were few studies included, as well as high heterogeneity and small sample sizes. The current study's findings are anomalous with previous research with the ALSPAC data, especially work examining depression and systemic inflammation. For example, previous cross-sectional research using the ALSPAC cohort found a linear, dose-response relationship between serum CRP and generalized anxiety disorder at age 16 years (Khandaker et al., 2016). Longitudinal analyses of the data have also shown that serum IL-6 at age 9 years predicted depression symptoms of diurnal mood variation, concentration difficulties, fatigue, and sleep disturbances at age 18 years (Chu et al., 2019), however, after controlling for confounders, there was no association between CRP at age 9 years and depression symptoms at age 18 years. Large-scale research in adults, using UK Biobank data, observed a causal relationship between both inflammatory markers, CRP and IL-6, and depression (Khandaker et al., 2020). Similar research using UK Biobank data found an association between CRP and anxiety, however this association was attenuated when controlling for depression and health-related factors (Kennedy and Niedzwiedz, 2022).

The inconsistency in findings between the current study and previous research may be explained by several potential factors. One potential explanation may be that CRP concentrations may be elevated in line with symptom duration in addition to symptom severity, and symptom duration is a factor that cannot be assessed using the current dataset. Another potential factor may lie in which inflammatory biomarkers are used to measure systemic inflammation. The present study only included one inflammatory marker at age 17.5, CRP. Future studies would benefit from including more inflammatory markers, allowing for a more comprehensive examination of the relationship between mental health and systemic inflammation. Further, despite anxiety and depressive disorders having heterogenous presentations (Buch and Liston, 2021; Drzewiecki and Fox, 2024) most studies examining the relationship between anxiety and/or depression and systemic inflammation have broadly operationalised depression or anxiety, with few examining specific symptoms or symptom clusters. The use of different mental health psychometric measures, which measure different aspects of symptom presentation, may account for differences in findings across studies exploring the relationship between mental health and inflammation.

### 4.2 Mental health, systemic CRP, and pain symptoms

A notable observation in the current study is that elevated CRP was associated with pain-type somatic symptoms, independent of mental health status, indicating that there may be inflammatory processes underlying pain-type somatic symptoms, independent of anxiety or depression symptom. It is likely that the associations between inflammation and mental health is symptom-specific, such that different facets of anxiety and depression relate differently to different markers of inflammation, as has been reported for depression in other studies. For example, Frank et al. (2021) completed a review of systemic inflammation and specific symptoms of depression, and while their review focused on non-pain related somatic symptoms of depression (changes in appetite, feeling everything was an effort, loss of energy, sleep problems), they observed higher serum and plasma CRP concentrations associated with those somatic symptoms of depression. Similar associations have been observed between serum and plasma CRP and IL-6, and somatic depression symptoms, including, appetite changes and sleep disturbances (Frank et al., 2021; Jokela et al., 2016; Moriarity et al., 2023), and somatic anxiety symptoms (plasma CRP; Duivis et al., 2013). Bai et al. (2014) observed that elevated levels of sP-selectin, a protein which plays a role in the inflammatory response, were associated with pain-type somatic symptoms and non-pain-type somatic symptoms among individuals with depression.

A significant, negative association was observed between the interaction term snxiety∗depression and pain-type somatic symptoms. This may indicate a ceiling effect whereby pain symptoms reach a saturation point, beyond which increasing psychological distress does not continue to contribute to pain linearly. Alternatively, individuals experiencing high levels of comorbid anxiety and depression symptoms may exhibit different symptom profiles which are less associated with pain experience or different types of pain experience (de Heer et al., 2014), or may engage in different health behaviours which may themselves influence pain (Nicolson et al., 2009). Further research is needed to explore the nature of comorbid anxiety and depression and its associations with pain.

Further research to delineate the specific inflammatory processes and specific aspects of mental health, and physical health may provide more clarity in the treatment of both mental health conditions and pain symptoms. For example, the findings of the current study may indicate that some pain symptoms, which may be regarded as medically unexplained, can be interpreted as being associated with mental health conditions, even when they may have distinct biological underpinnings. To this end, there is a risk that in some instances diagnostic overshadowing may occur, in that pain symptoms may be misinterpreted as being primarily associated with mental health symptoms, leading to the under-recognition of potential underlying pathophysiological processes and health conditions (Jones et al., 2008; Molloy et al., 2023). This highlights the importance of exercising caution when selecting mental health screening tools, as some widely used measures are heavily weighted towards somatic or physical symptoms, which may blur the distinction between psychological distress and symptoms of physical illness/underlying pathophysiological processes (Wagner et al., 2012). Notably, while the effect of CRP on pain was smaller than that of anxiety in this samples (*β* = .12 vs. *β* = .20), it remained a statistically significant and independent predictor of pain symptoms. This is important, as anxiety is widely accepted in clinical settings as a contributor to somatic symptom burden. CRP's predictive value, though more modest, suggests that systemic inflammation may too be a contributor. Misinterpretation of the aetiology of pain symptoms is likely to affect treatment decisions, and overreliance on mental health conditions as a direct explanatory factor may result in pain symptoms not being treated appropriately, as has historically been the case with conditions such as fibromyalgia and myalgic encephalomyelitis/chronic fatigue syndrome (ME/CFS; Fitzcharles and Yunus, 2012; Keith and Aneez, 2016).

Alternative explanations, which could be further investigated in future longitudinal studies, include the possibility that pain-type somatic symptoms associated with systemic inflammation may present earlier than cognitive/affective symptoms of anxiety and depression. In this way, such symptoms may in effect play the role of the “canary in the coal mine” for emerging mental health conditions, offering potential opportunities for earlier identification and intervention. Alternatively, the burden of unexplained, and un-treated pain symptoms, and their impact on quality of life may give rise to the development of anxiety or depression. While these hypotheses remain speculative, and cannot be explored in the current study, at the very least, the findings of this study indicate that further exploration into underlying immunological processes of pains symptoms which may or may not be associated with mental health symptoms is warranted.

### 4.3 Implications/future directions/limitations

If some individuals' experience of pain symptoms are indeed being misattributed to mental health conditions, this may be highlighting the occurrence of misdiagnosis and diagnostic overshadowing. This has historically been the case for conditions like ME/CFS and fibromyalgia, which were long misunderstood and frequently misclassified as manifestations or presentations of mental health (Ercolani et al., 1994; Fitzcharles and Yunus, 2012; Keith and Aneez, 2016). ME/CFS and fibromyalgia, can present similarly to somatic symptoms of depression and anxiety, such as fatigue, pain, immunological symptoms (sore throat and tender lymph nodes), sleep disfunction, and whose severity can wax and wane according to triggers such as energy expenditure, and stress (Gomez-Arguelles et al., 2022; Nacul et al., 2020). While the pathophysiology of both ME/CFS and fibromyalgia are still not fully understood, there are neuroendocrine, neuroimmune and inflammatory processes underlying both conditions (Cortes Rivera et al., 2019; Siracusa et al., 2021). Heightened levels of circulating inflammatory cytokines have been observed in both ME/CFS (CRP, TNF-α, interleukin-2, interleukin-4, and transforming growth factor-β; Groven et al., 2019; Strawbridge et al., 2019) and fibromyalgia (IL-6 and Interleukin-8; Yepez et al., 2022). ME/CFS, fibromyalgia, and pain-type somatic symptoms may share similar changes or dysfunction in neurotransmitters such as glutamate, GABA, serotonin and norepinephrine, and/or changes in neural pain pathways such as in the hypothalamus, amygdala, insular cortex, and cingulated cortex, perhaps influenced by systemic and central inflammation (Han and Pae, 2015; Siracusa et al., 2021; Yepez et al., 2022). As such, pain symptoms which are thought to be "medically unexplained” in nature, or attributed to mental health conditions should be considered as potential indicators of underlying pathophysiological processes, and disease. The findings of the current study raise the possibility that subtle biological mechanisms underpinning these pain symptoms, such as systemic inflammation, may sometimes be present in the absence of mental health symptoms, and that they may not be routinely detected in clinical settings. Although the effect of CRP was modest in this sample, it's independent association with pain symptoms underscores the value of further exploring non-psychological contributors to pain in adolescents.

A more thorough understanding of the pathophysiology of pain symptoms, either in association with, or independent of mental health conditions may present avenues for treatment including stand-alone or adjunct medications, and inform behaviour-changes which could play an important role in improving health outcomes and quality of life. For example, the use of anti-inflammatory medications such as non-steroidal anti-inflammatories (Köhler-Forsberg et al., 2019), cytokine inhibitors (Felger, 2019), or antidepressants which reduce the levels of serum CRP (Jha et al., 2017) have been proposed as potential adjunct or monotherapies for individuals with immuno-metabolic depression, and in the same way may serve as potential pharmacotherapies for individuals who present with pain symptoms, with or without anxiety and/or depression. Other interventions, including exercise and dietary changes have been shown to reduce systemic inflammation (e.g. Donoso et al., 2022; Zheng et al., 2019), and through their anti-inflammatory effects may improve pain symptoms.

The cross-sectional nature of this study is a limitation which prevents us from drawing conclusions regarding causality. In addition, associations were explored during adolescence; future studies exploring the associations at different developmental stages, for example young and older adulthood, would increase the generalisability of the findings. The sample was 96% White British, limiting it's generalisability to other ethnicities. Further to this, ethnic differences in CRP levels have been observed, with a systematic review showing significant differences remained after adjusting for covariates (Nazmi and Victora, 2007), but a recent large-scale UK Biobank study observed that associations between ethnicity and CRP were almost completely attenuated when differences in socioenvironmental exposures (e.g. racial discrimination, poverty) and health outcomes were accounted for (Nagar et al., 2021). It's also worth noting that based on the data available from the ALSPAC dataset, the current study included only one inflammatory marker, CRP, while other pro- and anti-inflammatory molecules (such as IL-6, TNF-α, sP-selectin) have been associated with both anxiety and/or depression (e.g. Frank et al., 2021) and somatic pain (e.g. Bai et al., 2014).

The current study used an unvalidated scale for pain-type somatic symptoms, deriving the scale by summing the severity scores of 7 pain symptoms from the CIS-R Somatic Symptom scale (Lewis, 1994). Future studies would benefit from using a validated pain scale, be it one that focuses specifically on somatic symptoms or one that explores general pain symptoms. Finally, apart from diabetes, diagnoses of autoimmune or inflammatory diseases were not available from the dataset and as such could not be excluded. However, the prevalence of autoimmune disorders among those under 18 years of age is low, ranging from 1 to 3% for Hashimoto thyroiditis to .01% for Graves Disease (Grasso and Chiarelli, 2024), and in this way the potential impact on the findings is likely minimal. Indeed, if there are some participants in the sample with undiagnosed autoimmune or inflammatory disorders that contributed to pain-type somatic symptoms, this would still represent an association between CRP and pain symptoms that is independent of anxiety or depression symptoms.

While little research has been carried out exploring the associations between pain and peripheral cytokines, a recent review of studies exploring cytokines in cerebrospinal fluid (CSF) observed that in at least half of the studies, 21 cytokines were upregulated in the presence of chronic pain compared to controls, but little evidence for correlations between CSF cytokines and pain intensity (Rosenström et al., 2024). While further research is warranted exploring cytokine levels in CSF, it would also be advantageous for further studies to explore the associations between peripheral (e.g. blood serum) cytokines and pain as the means of obtaining these samples is less invasive for the participant, and less costly. Further to this, delineating the relationship between peripheral cytokines and different types of pain, including nociceptive, neuropathic, and nociplastic pain would also be valuable. As mentioned above, elucidating the relationship between peripheral cytokines and pain could not only advance our understanding of the pathophysiological mechanisms underlying pain, but also inform the development of clinically-feasible and -acceptable biomarkers, and ultimately guide targeted interventions. Further studies including these biomarkers would continue to build the body of evidence for immune-mediated pain-type somatic symptoms, and likely identify biological markers which could be investigated via blood tests, allowing for the identification of individuals who would benefit most from anti-inflammatory pharmacotherapies, or other anti-inflammatory interventions.

## Funding

This research was supported by the Irish Research Council under award number GOIPG/2019/4404.

## CRediT authorship contribution statement

**J. Cooney-Quane:** Conceptualization, Data curation, Formal analysis, Methodology, Visualization, Writing – original draft, Writing – review & editing. **D.S. Thomas:** Formal analysis, Writing – original draft, Writing – review & editing. **Y.M. Nolan:** Conceptualization, Supervision, Writing – original draft, Writing – review & editing. **S. Dockray:** Conceptualization, Formal analysis, Supervision, Writing – original draft, Writing – review & editing.

## Declaration of competing interest

The authors declare the following financial interests/personal relationships which may be considered as potential competing interests: Professor Yvonne Nolan- Given her role on the Editorial Board, had no involvement in the peer review of this article and had no access to information regarding its peer review. Full responsibility for the editorial process for this article was delegated to another journal editor. If there are other authors, they declare that they have no known competing financial interests or personal relationships that could have appeared to influence the work reported in this paper.

## Data Availability

The authors do not have permission to share data.
