# Supplementary material for: C-reactive protein is associated with pain-type somatic symptoms independent of mental health symptoms in adolescents: Evidence from the ALSPAC study
Source: Brain Behav Immun Health. 2025 Aug 5;48:101082. doi: 10.1016/j.bbih.2025.101082 (PMC12356028; doi:10.1016/j.bbih.2025.101082)
Supplement: Multimedia component 1 [file mmc1.docx]

**Supplementary Materials**

**Appendix A**

**Table A.1.**

| *Missing Values* | | |
| --- | --- | --- |
| Characteristic | Valid | Missing |
| Participants*, n* (%) | 2877 (100) | 0 (0) |
| Sex*, n* (%) | 2877 (100) | 0 (0) |
| Age*, n* (%) | 2877 (100) | 0 (0) |
| Ethnicity*, n* (%) | 2607 (90.6) | 270 (9.4) |
| Mother’s Education, *n* (%) | 2638 (91.7) | 239 (8.3) |
| Paternal Social Class, *n* (%) | 2436 (84.7) | 441 (15.3) |
| Mother’s Age at Delivery, *n* (*%*) | 2876 (100.0) | 1 (.0) |
| BMI*, n* (%) | 2835 (98.5) | 42 (1.5) |
| CRP*, n* (%) | 2877 (100) | 0 (0) |
| Anxiety Score*, n* (%) | 2679 (93.1) | 198 (6.9) |
| Depression Score*, n* (%) | 2687 (93.4) | 190 (6.6) |
| Pain-Type Somatic Symptom Score*, n* (%) | 2682 (93.2) | 195 (6.8) |
| Infection Present in Last 3 weeks, *n* (%) | 2589 (90.0) | 288 (10) |
| Daily Smoking*, n* (%) | 2490 (86.5) | 387 (13.5) |
| Alcohol Consumption*, n* (%) | 2484 (86.3) | 393 (13.7) |
| Substance Use*, n* (%) | 2465 (85.7) | 412 (14.3) |
| Valid, n (%; listwise) | 2339 (81.30) |  |

Abbreviations: BMI, body mass index; CRP, C-reactive protein; SD, standard deviation

**Appendix B**

CIS-R Depression Score Symptoms

Calculation of **Depression** symptom score, score one for each of:

• Unable to enjoy or take an interest in things as much as usual in past week.

• Felt sad, miserable or depressed/unable to enjoy or take an interest in things on four days or more in the past week.

• Felt, sad, miserable or depressed/unable to enjoy or take an interest in things for more than three hours in total on any day in past week.

• When sad, miserable or depressed you did not become happier when something nice happened, or when in company.

Calculation of **Fatigue** symptom score, score one for each of:

• Felt tired for four days or more in the past seven days.

• Felt tired for more than three hours in total on any day in past week.

• Felt so tired that you’ve had to push yourself to get things done on at least one occasion during the past week.

• Felt tired when doing things you enjoy or used to enjoy at least once during past week.

Calculation of **Concentration** symptom score, score one for each of:

• Noticed problems with concentration/memory for four days or more in the past week.

• Could not always concentrate on at least one of: TV programme, read a newspaper article or talk to someone, without mind wandering in past week.

• Problems with concentration actually stopped you from getting on with things you used to do or would like to do.

• Forget something important in past seven days.

Calculation of **Sleep Problems** symptom score, score for each item is provided in brackets:

• Had problems with sleep for four nights or more out of past seven (1).

• Spent between 15 minutes and one hour (1), or more than one hour (2) trying to get to sleep on the night with the least sleep (1).

• Four nights or more spent at least 3 hours trying to get to sleep (1)

Calculation of **Depressive Thoughts** symptom score, score one for each of:

• Felt guilty or blamed yourself when things went wrong when it hasn’t been your fault sometimes or often in past seven days.

• Felt that you are not as good as other people during past week.

• Felt hopeless, for instance about your future, during the past seven days.

• Felt that life isn’t worth living in past week, sometimes or always.

• Thought of harming yourself in the past week.

**Appendix C**

**Table C.1.**

| *Exclusion Criteria* |  |  | | | | |
| --- | --- | --- | --- | --- | --- | --- |
| Characteristic | | | Total (%) | Missing (%) | Yes (valid %) | No (valid %) |
| PLIKS-Q – Met Diagnosis for Psychotic Disorder, n (%) | | | 3095 (94.2) | 189 (5.8) | 28 (0.9) | 3067 (99.1) |
| Bleeding / Clotting Disorder, n (%) | | | 3250 (99) | 34 (1.0) | 6 (0.2) | 3244 (99.8) |
| Diabetes, n (%) | | | 3172 (96.6) | 112 (3.4) | 10 (0.3) | 3162 (99.7) |
| Hypertension, n (%) | | | 3172 (96.6) | 112 (3.4) | 24 (0.8) | 3148 (99.2) |
| High Cholesterol, n (%) | | | 3171 (96.6) | 113 (3.4) | 5 (0.2) | 3166 (99.8) |
| Vascular Disease, n (%) | | | 3170 (96.5) | 114 (3.5) | 27 (.9) | 3143 (99.1) |

**Appendix D**

*Table D.1. Unadjusted Regressions of Associations Between Pain-Type Somatic Symptoms, CRP, and Mental Health*

| Variable | *B* | *95% CI* | *SE* | *Beta* | *df* | *F* | *R2* | *R2(adj)* | *R2 Δ* |
| --- | --- | --- | --- | --- | --- | --- | --- | --- | --- |
| Step 1 |  |  |  |  |  |  |  |  |  |
| (Constant) | 1.39 | [.51, 2.27] | .45 | <-.01** |  |  |  |  |  |
| Ethnicity | .42 | [-.11, .95] | .27 | .03 |  |  |  |  |  |
| Sex | .85 | [.64, 1.07] | .11 | .17*** |  |  |  |  |  |
| Paternal Social Class | <-.01 | [-.10, .09] | .05 | <-.01 |  |  |  |  |  |
| Mums Highest Education | -.05 | [-.15, .05] | .05 | -.02 |  |  |  |  |  |
| Maternal Age at Delivery (grouped) | <-.01 | [-.11, .10] | .05 | <-.01 |  |  |  |  |  |
|  |  |  |  |  | 5, 2099 | 12.80 | .03 | .03 | .03 |
| Step 2 |  |  |  |  |  |  |  |  |  |
| (Constant) | 1.47 | [-.90, 3.84] | 1.21 | <-.01 |  |  |  |  |  |
| Ethnicity | .45 | [-.06, .97] | .26 | .04 |  |  |  |  |  |
| Sex | .83 | [.62, 1.04] | .11 | .16*** |  |  |  |  |  |
| Paternal Social Class | -.03 | [-.12, .07] | .05 | -.01 |  |  |  |  |  |
| Mums Highest Education | -.06 | [-.15, .04] | .05 | -.03 |  |  |  |  |  |
| Maternal Age at Delivery (grouped) | <.01 | [-.10, .10] | .05 | <.01 |  |  |  |  |  |
| BMI (log10) | 1.38 | [-.19, 2.95] | .80 | .04 |  |  |  |  |  |
| Alcohol Consumption | .07 | [.04, .10] | .02 | .10*** |  |  |  |  |  |
| Substance Use (3months) | .20 | [.05, .35] | .07 | .06** |  |  |  |  |  |
| Daily Smoking | .04 | [.01, .07] | .01 | .06* |  |  |  |  |  |
| Infection (last 3 weeks) | -1.27 | [-1.56, -.97] | .15 | -.18*** |  |  |  |  |  |
|  |  |  |  |  | 5, 2094 | 20.75 | .09 | .09 | .06 |
| Step 3 |  |  |  |  |  |  |  |  |  |
| (Constant) | 1.41 | [-.77, 3.59] | 1.11 | <-.01 |  |  |  |  |  |
| Ethnicity | .41 | [-.07, .88] | .24 | .03 |  |  |  |  |  |
| Sex | .45 | [.26, .65] | .10 | .09*** |  |  |  |  |  |
| Paternal Social Class | -.03 | [-.11, .05] | .04 | -.01 |  |  |  |  |  |
| Mums Highest Education | -.05 | [-.14, .04] | .05 | -.02 |  |  |  |  |  |
| Maternal Age at Delivery (grouped) | .02 | [-.07, .11] | .05 | <.01 |  |  |  |  |  |
| BMI (log10) | 1.08 | [-.36, 2.52] | .74 | .03 |  |  |  |  |  |
| Alcohol Consumption | .06 | [.03, .09] | .01 | .08*** |  |  |  |  |  |
| Substance Use (3months) | .06 | [-.07, .20] | .07 | .02 |  |  |  |  |  |
| Daily Smoking | .02 | [-.01, .05] | .01 | .03 |  |  |  |  |  |
| Infection (last 3 weeks) | -1.10 | [-1.37, -.83] | .14 | -.15*** |  |  |  |  |  |
| Anxiety score | .31 | [.12, .49] | .10 | .09** |  |  |  |  |  |
| Depression Score | .26 | [.23, .29] | .01 | .38*** |  |  |  |  |  |
| Anxiety*Depression | -.10 | [-.18, -.03] | .04 | -.08** |  |  |  |  |  |
|  |  |  |  |  | 3, 2091 | 48.61 | .23 | .23 | .14 |

^*^p < .05. ^**^p < .01. ^***^p < .001, CI = Confidence Interval for *B*

*Table D.2. Unadjusted Regressions of Associations Between* *Mental Health and CRP*

| Variable | *B* | *95% CI* | *SE* | *Beta* | *df* | *F* | *R2* | *R2(adj)* | *R2 Δ* |
| --- | --- | --- | --- | --- | --- | --- | --- | --- | --- |
| Step 1 |  |  |  |  |  |  |  |  |  |
| (Constant) | -.43 | [-.59, -.27] | .08 | <-.01*** |  |  |  |  |  |
| Ethnicity | -.01 | [-.11, .09] | .05 | <-.01 |  |  |  |  |  |
| Sex | .16 | [.12, .20] | .02 | .17*** |  |  |  |  |  |
| Paternal Social Class | .02 | [.01, .04] | <.01 | .06* |  |  |  |  |  |
| Mums Highest Education | -.01 | [-.03, .01] | <.01 | -.03 |  |  |  |  |  |
| Maternal Age at Delivery (grouped) | <-.01 | [-.02, .02] | .01 | <-.01 |  |  |  |  |  |
|  |  |  |  |  | 5, 2099 | 14.83 | .03 | .03 | .03 |
| Step 2 |  |  |  |  |  |  |  |  |  |
| (Constant) | -2.92 | [-3.34, -2.50] | .21 | <-.01*** |  |  |  |  |  |
| Ethnicity | -.03 | [-.12, .06] | .05 | -.01 |  |  |  |  |  |
| Sex | .14 | [.10, .17] | .02 | .14*** |  |  |  |  |  |
| Paternal Social Class | .02 | [.00, .04] | <.01 | .05* |  |  |  |  |  |
| Mums Highest Education | <-.01 | [-.02, .01] | <.01 | -.02 |  |  |  |  |  |
| Maternal Age at Delivery (grouped) | <.01 | [-.01, .03] | <.01 | .02 |  |  |  |  |  |
| BMI (log10) | 2.13 | [1.86, 2.41] | .14 | .31*** |  |  |  |  |  |
| Alcohol Consumption | <.01 | [-.00, .01] | <.01 | .02 |  |  |  |  |  |
| Substance Use (3months) | <.01 | [-.02, .04] | .01 | .01 |  |  |  |  |  |
| Daily Smoking | <.01 | [-.00, .01] | <.01 | .02 |  |  |  |  |  |
| Infection (last 3 weeks) | -.22 | [-.27, -.17] | .03 | -.17*** |  |  |  |  |  |
|  |  |  |  |  | 5, 2094 | 39.59 | .16 | .16 | .13 |
| Step 3 |  |  |  |  |  |  |  |  |  |
| (Constant) | -2.91 | [-3.33, -2.49] | .21 | <-.01*** |  |  |  |  |  |
| Ethnicity | -.03 | [-.12, .06] | .05 | -.01 |  |  |  |  |  |
| Sex | .14 | [.10, .17] | .02 | .15*** |  |  |  |  |  |
| Paternal Social Class | .02 | [.00, .04] | <.01 | .05* |  |  |  |  |  |
| Mums Highest Education | <-.01 | [-.02, .01] | <.01 | -.02 |  |  |  |  |  |
| Maternal Age at Delivery (grouped) | <.01 | [-.01, .03] | <.01 | .02 |  |  |  |  |  |
| BMI (log10) | 2.13 | [1.85, 2.41] | .14 | .31*** |  |  |  |  |  |
| Alcohol Consumption | <.01 | [-.00, .01] | <.01 | .02 |  |  |  |  |  |
| Substance Use (3months) | <.01 | [-.02, .04] | .01 | .01 |  |  |  |  |  |
| Daily Smoking | <.01 | [-.00, .01] | <.01 | .02 |  |  |  |  |  |
| Infection (last 3 weeks) | -.22 | [-.27, -.17] | .03 | -.17*** |  |  |  |  |  |
| Anxiety score | -.04 | [-.07, -.00] | .02 | -.06* |  |  |  |  |  |
| Depression Score | <.01 | [-.01, .01] | <.01 | <.01 |  |  |  |  |  |
| Anxiety*Depression | <.01 | [-.01, .02] | <.01 | .03 |  |  |  |  |  |
|  |  |  |  |  | 3, 2091 | 30,78 | .16 | .16 | <.01 |

^*^p < .05. ^**^p < .01. ^***^p < .001, CI = Confidence Interval for *B*

*Table D.3. Unadjusted Regressions of Associations Between Pain-Type Somatic Symptoms, CRP, and Mental Health*

| Variable | *B* | *95% CI* | *SE* | *Beta* | *df* | *F* | *R2* | *R2(adj)* | *R2 Δ* |
| --- | --- | --- | --- | --- | --- | --- | --- | --- | --- |
| Step 1 |  |  |  |  |  |  |  |  |  |
| (Constant) | 1.39 | [.51, 2.27] | .45 | <-.01** |  |  |  |  |  |
| Ethnicity | .42 | [-.11, .95] | .27 | .03 |  |  |  |  |  |
| Sex | .85 | [.64, 1.07] | .11 | .17*** |  |  |  |  |  |
| Paternal Social Class | <-.01 | [-.10, .09] | .05 | <-.01 |  |  |  |  |  |
| Mums Highest Education | -.05 | [-.15, .05] | .05 | -.02 |  |  |  |  |  |
| Maternal Age at Delivery (grouped) | <-.01 | [-.11, .10] | .05 | <-.01 |  |  |  |  |  |
|  |  |  |  |  | 5, 2099 | 12.80 | .03 | .03 | .03 |
| Step 2 |  |  |  |  |  |  |  |  |  |
| (Constant) | 1.47 | [-.90, 3.84] | 1.21 | <-.01 |  |  |  |  |  |
| Ethnicity | .45 | [-.06, .97] | .26 | .04 |  |  |  |  |  |
| Sex | .83 | [.62, 1.04] | .11 | .16*** |  |  |  |  |  |
| Paternal Social Class | -.03 | [-.12, .07] | .05 | -.01 |  |  |  |  |  |
| Mums Highest Education | -.06 | [-.15, .04] | .05 | -.03 |  |  |  |  |  |
| Maternal Age at Delivery (grouped) | <.01 | [-.10, .10] | .05 | <.01 |  |  |  |  |  |
| BMI (log10) | 1.38 | [-.19, 2.95] | .80 | .04 |  |  |  |  |  |
| Alcohol Consumption | .07 | [.04, .10] | .02 | .10*** |  |  |  |  |  |
| Substance Use (3months) | .20 | [.05, .35] | .07 | .06** |  |  |  |  |  |
| Daily Smoking | .04 | [.01, .07] | .01 | .06* |  |  |  |  |  |
| Infection (last 3 weeks) | -1.27 | [-1.56, -.97] | .15 | -.18*** |  |  |  |  |  |
|  |  |  |  |  | 5, 2094 | 20.75 | .09 | .09 | .06 |
| Step 3 |  |  |  |  |  |  |  |  |  |
| (Constant) | 1.41 | [-.77, 3.59] | 1.11 | <-.01 |  |  |  |  |  |
| Ethnicity | .41 | [-.07, .88] | .24 | .03 |  |  |  |  |  |
| Sex | .45 | [.26, .65] | .10 | .09*** |  |  |  |  |  |
| Paternal Social Class | -.03 | [-.11, .05] | .04 | -.01 |  |  |  |  |  |
| Mums Highest Education | -.05 | [-.14, .04] | .05 | -.02 |  |  |  |  |  |
| Maternal Age at Delivery (grouped) | .02 | [-.07, .11] | .05 | <.01 |  |  |  |  |  |
| BMI (log10) | 1.08 | [-.36, 2.52] | .74 | .03 |  |  |  |  |  |
| Alcohol Consumption | .06 | [.03, .09] | .01 | .08*** |  |  |  |  |  |
| Substance Use (3months) | .06 | [-.07, .20] | .07 | .02 |  |  |  |  |  |
| Daily Smoking | .02 | [-.01, .05] | .01 | .03 |  |  |  |  |  |
| Infection (last 3 weeks) | -1.10 | [-1.37, -.83] | .14 | -.15*** |  |  |  |  |  |
| Anxiety score | .31 | [.12, .49] | .10 | .09** |  |  |  |  |  |
| Depression Score | .26 | [.23, .29] | .01 | .38*** |  |  |  |  |  |
| Anxiety*Depression | -.10 | [-.18, -.03] | .04 | -.08** |  |  |  |  |  |
|  |  |  |  |  | 3, 2091 | 48.61 | .23 | .23 | .14 |
| Step 4 |  |  |  |  |  |  |  |  |  |
| (Constant) | 3.45 | [1.20, 5.71] | 1.15 | <-.01** |  |  |  |  |  |
| Ethnicity | .42 | [-.05, .90] | .24 | .03 |  |  |  |  |  |
| Sex | .36 | [.16, .56] | .10 | .07*** |  |  |  |  |  |
| Paternal Social Class | -.04 | [-.12, .04] | .04 | -.02 |  |  |  |  |  |
| Mums Highest Education | -.04 | [-.13, .05] | .04 | -.02 |  |  |  |  |  |
| Maternal Age at Delivery (grouped) | .01 | [-.08, .11] | .05 | <.01 |  |  |  |  |  |
| BMI (log10) | -.42 | [-1.92, 1.09] | .77 | -.01 |  |  |  |  |  |
| Alcohol Consumption | .05 | [.03, .08] | .01 | .08*** |  |  |  |  |  |
| Substance Use (3months) | .06 | [-.08, .19] | .07 | .02 |  |  |  |  |  |
| Daily Smoking | .02 | [-.01, .05] | .01 | .03 |  |  |  |  |  |
| Infection (last 3 weeks) | -.94 | [-1.22, -.67] | .14 | -.13*** |  |  |  |  |  |
| Anxiety score | .33 | [.15, .52] | .09 | .09*** |  |  |  |  |  |
| Depression Score | .26 | [.23, .29] | .01 | .38*** |  |  |  |  |  |
| Anxiety*Depression | -.11 | [-.18, -.04] | .04 | -.08** |  |  |  |  |  |
| CRP (log10) | .70 | [.48, .92] | .11 | .13*** |  |  |  |  |  |
|  |  |  |  |  | 1, 2090 | 48.73 | .25 | .24 | .01 |

^*^p < .05. ^**^p < .01. ^***^p < .001, CI = Confidence Interval for *B*

**Appendix E**

**Table E.1.**

| *Mann-Whitney U Tests according to Sex* | | | | | | |
| --- | --- | --- | --- | --- | --- | --- |
|  | Males | Females |  |  |  |  |
|  | *Md* | *Md* | *U* | *z* | *p* | *r* |
| Anxiety Score | 0.00 | .00 | 821823 | -5.96 | <.001 | -.11.5 |
| Depression Score | 1.00 | 2.00 | 710548 | -9.73 | <.001 | - 0.19 |
| Pain-Type Somatic Symptom Score | 2.00 | 3.00 | 726599 | -8.65 | <.001 | -0.17 |
| CRP mg/l | 0.45 | 0.67 | 836396 | -8.85 | <.001 | -0.16 |
| BMI | 21.66 | 21.98 | 962811 | -1.87 | .06 | -0.04 |
| Daily Smoking | 0.00 | 0.00 | 769561 | -.48 | .63 | -0.01 |
| Alcohol Consumption | 4.00 | 4.00 | 714038 | -3.19 | .001 | -0.06 |
| Substance Use | 0.00 | 0.00 | 740075 | -1.45 | .15 | -0.03 |
